# Mutation status and prognostic values of *KRAS*, *NRAS*, *BRAF* and *PIK3CA* in 353 Chinese colorectal cancer patients

**DOI:** 10.1038/s41598-018-24306-1

**Published:** 2018-04-17

**Authors:** Fang Guo, Hai Gong, Huanhuan Zhao, Jing Chen, Yiming Zhang, Lihua Zhang, Xin Shi, Aifeng Zhang, Hui Jin, Jianqiong Zhang, Youji He

**Affiliations:** 1 Department of Pathogenic Biology and Immunology, Medical School of Southeast University, Nanjing, Jiangsu China; 2 Department of Colorectal Surgery, Jiangyin People’s Hospital affiliated to Southeast University, Jiangyin, Jiangsu China; 3 Institute of Life Sciences, Southeast University, Nanjing, Jiangsu China; 4 Department of Pathology, Zhongda Hospital Affiliated to Southeast University, Nanjing, Jiangsu China; 5 Department of General Surgery, Zhongda Hospital Affiliated to Southeast University, Nanjing, Jiangsu China; 6 Department of Epidemiology, School of Public Health, Southeast University, Nanjing, Jiangsu China; 7 Key Laboratory of Developmental Genes and Human Disease, Ministry of Education, Department of Microbiology and Immunology, Medical School, Southeast University, Nanjing, Jiangsu China; 8 Jiangsu Key Laboratory of Molecule Imaging and Functional Imaging, Zhongda Hospital, Medical School, Southeast University, Nanjing, Jiangsu China

## Abstract

Mutations in *KRAS* exon 2, *BRAF* and *PIK3CA* are commonly present in colorectal cancer (CRC) worldwide, but few data about *RAS* mutations outside *KRAS* exon 2 are available for Chinese CRCs. We, therefore, determined the mutation frequencies and prognostic values of *KRAS* exon 2, 3 and 4, *NRAS* exon 2 and 3, *PIK3CA* exon 9 and 20, and *BRAF* exon 15 by PCR and direct sequencing in 353 CRC patients from two Chinese clinical centers. *KRAS* exon 2, *BRAF*, *PIK3CA* mutations were identified in 42.2%, 4.5%, 12.3% of the cases, respectively. We found “rare mutations” in *RAS* genes in nearly 14% of CRCs-i.e., in almost a quarter (24.0%) of *KRAS* exon 2 wild type CRCs, including 2.3% in *KRAS* exon 3, 8.2% in *KRAS* exon 4 and 3.4% in *NRAS*. Stage I-III patients with *PIK3CA* or *NRAS* mutations developed more distant metastases (3-year risk in *PIK3CA* mutated and wild type patients: 23.3% vs 11.5%, P = 0.03; multivariate Hazard ratio (HR) = 3.129, P = 0.003; 3-year risk in *NRAS* mutated and wild type patients: 40.0% vs 12.2%, P = 0.012; multivariate HR = 5.152, P = 0.003). Our data emphasizes the importance of these novel molecular features in CRCs.

## Introduction

Colorectal cancer (CRC) is the third most common malignancy in the world. In the last few years, CRC has become the sixth most common malignancy and the fifth leading cause of malignancy-related mortality among the Chinese population^1^.

Activation of multiple signaling pathways of the Epidermal Growth Factor Receptor (EGFR), the RAS-RAF or the PI3K-PTEN-AKT pathways are considered the most common carcinogenic mechanisms in CRC^2^. Mutations of *RAS*, *BRAF* or *PIK3CA* cause constitutive activations in these two pathways. Mutations of *KRAS* exon 2, *BRAF* and *PIK3CA* are most common among CRCs with frequencies of 30–50%, 10–15% and 10–20%, respectively. *KRAS* exon 3 and 4 mutations contribute to a lower degree, only accounting for 1% and 4%^3^. *NRAS* mutations are found in about 3–5% and *HRAS* mutations were rare in previous studies^4,5^.

The *KRAS* exon 2 mutation was widely regarded as a predictor for anti-EGFR MoAbs resistance among CRCs^6^. Nevertheless, the majority of *KRAS* exon 2 wild-type (wt) patients fail to benefit from anti-EGFR MoAbs, implying the possibility that activating mutations in other *KRAS* exons or other genes may cause this resistance as well. Recently, a small number of clinical trials have shown that mutations in other *RAS* exons, such as mutations in *KRAS* exons 3 and 4, and *NRAS* can also predict resistance to anti-EGFR MoAbs^7–9^. Although many clinical data showed that *BRAF* or *PIK3CA* mutations were likely to be associated with anti-EGFR MoAbs resistance^10^, their predictive role is still controversial.

The benefits of individual genetic profiling for the selection of therapy have been proven in clinical use, but studies concerning the mutation frequencies and efficacy of targeted therapies were mostly presented in Western countries and few data are available for China^11^, especially for mutations in *KRAS* exons 3 and 4, and *NRAS*, mainly because the frequency of such mutations was considered low in literature. Moreover, few studies on the prognostic role of these rare mutations are available among Chinese CRC patients, due to lack of follow-up information.

Distant metastasis is a major problem of stage I to III CRC patients after surgery, as it is associated with both high morbidity and mortality. Effective postoperative adjuvant treatment, such as chemotherapy and radiotherapy, can improve patient outcome. Usually such treatment decisions are based on patients’ prognostic features, including traditional clinicopathological features (such as TNM stage, histopathological differentiation grade, invasion to surrounding tissue, and number of lymph-node metastases), microsatellite instability (MSI), and DNA mismatch repair status. According to the National Comprehensive Cancer Network clinical practice guidelines in oncology prior to 2016, stage II and III patients who are assessed to have a poor prognosis, need postoperative chemotherapy, except those who have a high frequency of MSI (MSI-H)^12^. Although postoperative chemotherapy resulted in a reduction of distant metastasis, patients still had a lower survival rate^13^. These clinical features, therefore, do not seem to make an exact evaluation of tumor development, so that patients with similar features still reveal difference in survival: quite a few were assessed at low risk and did not receive adjuvant therapy, but developed metastases shortly after surgery; on the other hand, high risk patients endured a decrease in quality of life due to excessive treatment. This discrepancy suggests that patients with similar clinocopathologic characteristics harbor a different genetic biology that regulates their tumor development.

In the last decade, many studies showed that molecular genetic changes can be more accurate markers than clinicopathological features to evaluate the prognosis of cases with early and medium stage CRC. In a study of 450 patients with stage I to III colon cancer, for example, *PIK3CA* mutations predicted a poorer prognosis, but only among *KRAS* wild-type CRC patients^14^. *PIK3CA* mutation was also identified as an independent biomarker for local recurrence among stage I-III rectal cancers^15^. Other studies suggested that *BRAF* mutations confer a poorer prognosis on stage II to III colon cancers, but no conclusive prognostic significance for *KRAS* mutations could be reached among early and medium stage CRCs^16–18^. Only one study showed that *NRAS* mutations predicted a poor outcome for CRC patients with metastases^19^. Despite the inconsistencies in these studies, they all suggest that biological markers will make a precise assessment of patient outcome in stage I to III CRCs possible and can improve the selection of patients for adjuvant treatment after surgery.

Our previous study of 214 Chinese CRC patients^20^ reported the mutation status and the prognostic values of *KRAS* exon 2, *BRAF* and *PIK3CA*, respectively. However, we did not analyze the “rare mutation” status at other locations of the *KRAS* and *NRAS* genes. Furthermore, the cohort (214) was relatively small. We, therefore, recruited more patients from another clinical center to extend the cohort to 353 CRC patients. We further analyzed additional mutations (including *KRAS* mutations outside exon 2 and *NRAS* mutations) and investigated the relationship between mutations and the clinicopathological features. Furthermore, we collected patients’ follow-up information and determined whether a mutation may be used as prognostic biomarker.

## Materials and Methods

### Samples

**Figure 1:**
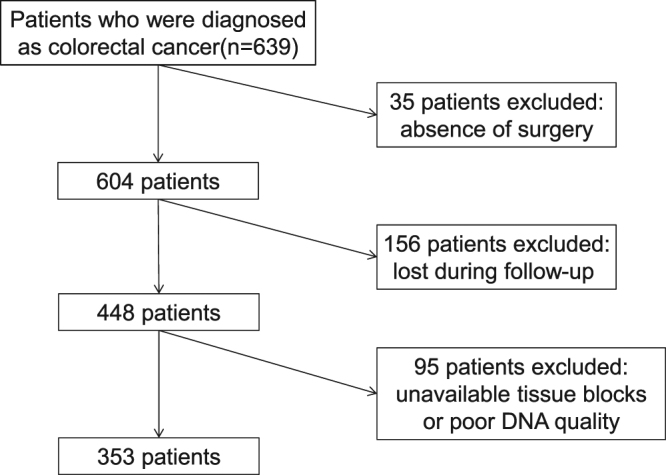
Selection of study population.

Between 2007 and 2012, we consecutively collected 436 CRC patients at Zhongda Hospital (Nanjing, China; the same patients from our previous study^20^) and 203 CRC patients at Jiangyin People’s Hospital (Jiangyin, China). Patients who did not undergo surgery (n = 35), were lost during the follow-up period (n = 156), had no tissue blocks available, or had poor DNA quality of the tumor sample (n = 95) were excluded. In total, 353 patients were included for genetic detection (Fig. 1). There was no difference in clinicopathological parameters between the in- and excluded patients (see Supplementary Table S1). All cases were diagnosed as CRC by two independent pathologists. No patients had accepted preoperative adjuvant treatment. The patients’ information is listed in Table 1. The collection of tissues and inclusion of patients were approved by the Institutional Ethics Committee of Zhongda Hospital and Jiangyin People’s Hospital. Written informed consent was obtained from all study subjects. The study was conducted according to the institutional guidelines and regulations set by Chinese law for the use of human material for research. The median follow-up for survivors was 33 months.

**Table 1:** Clinicopathological characteristics according to RAS-RAF/PI3K pathway gene mutation status in 353 (350) colorectal cancer patient.

		case	KRAS(exon2/3/4)			NRAS(exon2/3)			BRAF(exon15)			PIK3CA(exon9/20)*			PIK-pathway*		
		353(350)	No, n (%)	Yes, n (%)	p value	No, n (%)	Yes, n (%)	p value	No, n (%)	Yes, n (%)	p value	No, n (%)	Yes, n (%)	p value	No, n (%)	Yes, n (%)	p value
sex	Male	204 (202)	98(58.7)	106(57.0)	0.748^a^	201(58.9)	3(25.0)	**0.019** ^a^	194 (57.6)	10 (62.5)	0.696^a^	172 (56.6)	30 (65.2)	0.269^a^	79(59.8)	124(56.6)	0.553^a^
	Female	149 (148)	69 (41.3)	80(43.0)		140(41.1)	9(75.0)		143 (42.4)	6 (37.5)		132 (43.4)	16 (34.8)		53 (40.2)	95(43.4)	
Age			65.04	67.02	0.137^d^	65.96	69.5	0.335^d^	66.03	67.06	0.748^d^	66.4	64.37	0.298^d^	64.44	67.21	**0.040** ^d^
location	Colon	210(208)	106 (63.5)	104(55.9)	0.149^a^	203(59.5)	7(58.3)	1.000^b^	197 (58.5)	13 (81.3)	0.070^a^	173 (56.9)	35 (76.1)	**0.014** ^a^	79 (59.8)	129 (58.9)	0.862^a^
	Rectum	143(142)	61(36.5)	82 (44.1)		138 (40.5)	5 (41.7)		140 (41.5)	3 (18.8)		131 (43.1)	11(23.9)		53 (40.2)	90 (41.1)	
Differentiation	Well	44	23 (13.8)	21(11.3)	0.780^c^	42(12.3)	2(16.7)	0.484^c^	43 (12.8)	1 (6.3)	**0.030** ^c^	36 (11.8)	8 (17.4)	0.469^c^	18 (13.6)	26 (11.9)	0.965^c^
	moderate	274(272)	126 (75.4)	148(79.6)		265(77.7)	9(75.0)		264 (78.3)	10 (62.5)		239 (78.6)	33 (71.7)		102 (77.3)	170 (77.6)	
	Poor	12(11)	9(5.4)	3 (1.6)		12 (3.5)	0 (0)		9 (2.7)	3 (18.8)		9 (3.0)	2 (4.3)		6 (4.5)	6 (2.7)	
	Missing	23	9 (5.4)	14(7.5)		22 (6.5)	1 (8.3)		21 (6.2)	2 (12.5)		20 (6.6)	3 (6.5)		6 (4.5)	17 (7.8)	
Tumor diameter	<5cm	171(169)	79 (47.3)	92 (49.5)	0.579^a^	165 (48.4)	6(50.0)	0.936^a^	164 (48.7)	7 (43.8)	**0.676** ^a^	151 (49.7)	18 (39.1)	0.211^a^	62 (47.0)	108 (49.3)	0.583^a^
	>=5cm	179(178)	88 (52.7)	91 (48.9)		173 (50.7)	6(50.0)		170 (50.4)	9 (56.3)		151 (49.7)	27 (58.7)		70 (53.0)	108 (49.3)	
	Missing	3	0	3 (1.6)		3(0.9)	0(0)		3 (0.9)	0 (0)		2 (0.7)	1 (2.2)		0 (2.2)	3 (1.4)	
TNM stage	I	53(53)	21(12.6)	32(17.2)	0.752^c^	52(15.2)	1(8.3)	0.692^c^	53(15.7)	0()	**0.001** ^c^	43(14.1)	10(21.7)	0.122^c^	17(12.9)	36(16.4)	0.405^c^
	II	126(125)	69(41.3)	57(30.6)		121(35.5)	5(41.7)		123(36.5)	3(18.8)		108(35.5)	17(37.0)		56(42.4)	69(31.5)	
	III	126	53(31.7)	73(39.2)		122(35.8)	4(33.3)		120(35.6)	6(37.5)		109(35.9)	16(34.8)		42(31.8)	83(37.9)	
	IV	45(44)	22(13.2)	23(12.4)		43(12.6)	2(16.7)		38(11.3)	7(43.8)		41(13.5)	3(6.5)		15(11.4)	30(13.7)	
	Missing	3	2(1.2)	1(0.5)		3(0.9)	0(0)		3(0.9)	0(0)		3(1.0)	0(0)		2(1.5)	1(0.5)	
T	T1	5	2 (1.2)	3 (1.6)	0.197^c^	5 (1.5)	0	0.532^c^	5 (1.5)	0	**0.019** ^c^	5 (1.6)	0	0.816^c^	2 (1.5)	3 (1.4)	0.542^c^
	T2	69	28 (16.8)	41 (22.2)		68 (19.9)	1 (8.3)		69 (20.5)	0		57 (18.8)	12 (26.1)		24 (18.2)	45 (20.5)	
	T3	258(256)	125 (74.9)	133 (71.5)		247 (72.4)	11 (91.7)		244 (72.4)	14 (87.5)		226 (74.3)	30 (65.2)		96 (72.7)	161 (73.5)	
	T4	19(18)	10 (6.0)	9 (4.8)		19 (5.6)	0		17 (5.0)	2 (12.5)		14 (4.6)	4 (8.7)		8 (6.1)	10 (4.6)	
	Missing	2	2 (1.2)	0 (0)		2 (0.6)	0		2 (0.6)	0		2 (0.7)	0		2 (1.5)	0	
N	N(−)	189(188)	97(58.1)	92 (49.5)	0.102^a^	181 (53.1)	8 (66.7)	0.365^a^	186 (55.2)	3(18.8)	**0.004** ^a^	160 (52.6)	28(60.9)	0.317^a^	78 (59.1)	110 (50.2)	0.099^a^
	N(+)	162(160)	69 (41.3)	93 (50.0)		158(46.3)	4 (33.3)		149 (44.2)	13 (81.3)		142 (46.7)	18 ((39.1)		53 (40.2)	108 (49.3)	
	Missing	2	1 (0.6)	1(0.5)		2 (0.6)	0 (0)		2 (0.6)	0		2(0.7)	0 (0)		1 (0.8)	1 (0.5)	
M-synchronous	(−)	306(304)	144(86.2)	162(87.1)	0.818^a^	296(86.8)	10(83.3)	0.657^b^	297(88.1)	9(56.3)	**0.002** ^b^	261(85.9)	43(93.5)	0.180^a^	116(87.9)	188(85.8)	0.533^a^
	(+)	45(44)	22(13.2)	23(12.4)		43(12.6)	2(16.7)		38(11.3)	7(43.8)		41(13.5)	3(6.5)		15(11.4)	30(13.7)	
	Missing	2	1(0.6)	1(0.5)		2(0.6)	0(0)		2(0.6)	0		2(0.6)	0(0)		1(0.8)	1(0.5)	
M-metachronous	(−)	300(297)	141(84.4)	159(85.5)	0.782^a^	292(85.6)	8(66.7)	0.089^b^	290(86.1)	10(62.5)	**0.021** ^b^	262(86.2)	35(76.1)	0.075^a^	117(88.6)	181(82.6)	0.129^a^
	(+)	53	26(15.6)	27(14.5)		49(14.4)	4(33.3)		47(13.9)	6(37.5)		42(13.8)	11(23.9)		15(11.4)	38(17.4)	

^a^Chi-square test; ^b^Fisher exact test; ^c^Mann-Whitney test; ^d^t test. *DNA of three samples was not available for PIK3CA exon 20.

### DNA extraction

Genomic DNA was extracted from 5 sections of 10 μm thickness of macro-dissected formalin-fixed paraffin-embedded (FFPE) tumor samples, containing at least 50% tumor epithelium, as determined by two experienced pathologists in H&E-stained paraffin sections. The QIAmp DNA Mini Kits (Qiagen GmbH, Hilden, Germany) were used according to the manufacturer’s instructions.

### PCR and Direct sequencing

For each sample, mutations of *KRAS* exons 2, 3 and 4, *NRAS* exons 2 and 3, *PIK3CA* exons 9 and 20, and *BRAF* exon 15 were amplified by polymerase chain reaction (PCR). Amplification was performed for 30 cycles with the following settings: 95 °C for 4 min (only first cycle); 94 °C for 30 s, 55 °C for 30 s (1 min for the PIK3CA exon 9 and 20 mutations), and 72 °C for 1 min; the final extension cycle was carried out at 72 °C for 7 minutes (10 min for the PIK3CA exon 9 and 20 mutations). The presence of mutations was detected by direct sequencing at Beijing Genomic Institute (BGI, ABI 3730xL Genetic analyzer, Shenzhen, China), using the BigDye Terminator Cycle Sequencing kit (Applied Biosystems). Forward and reverse sequencing were carried out to confirm mutant PCR products. Primer information is listed in Supplementary Table S2.

### Statistical analyses

SPSS statistical software (version 18.0 for Windows, SPSS, Inc.) was used for statistical analyses. Categorical variables were compared by the chi-square or Fisher’s exact test; quantitative and ordered variables were compared by the Mann-Whitney test. Normally distributed variables were compared by Student’s t test. Metastasis time was defined as the period between surgery and the detection of a distant metastasis. Overall Survival (OS) was defined as the period between surgery and death of any cause or last follow-up visit. The Kaplan-Meier (KM) method and Log-rank tests were used to evaluate the time to diagnosis of metastases and survival.

To study variables associated with metastasis or survival, we first evaluated the variables (listed in Table 2) in a univariate Cox regression model. Variables with P < 0.1 were taken into a multivariate Cox regression model with stepwise backward elimination. A two-sided P value ≤ 0.05 was considered statistically significant.

**Table 2:** Analysis of distant metastasis in 305 CRC patients with TNM stage I to III by univariate and multivariate Cox regression analysis.

Variables	Univarite analysis		Multivarite analysis	
	HR(95% CI)	P	HR(95% CI)	P
Age		0.578		
<=65	1.0			
>65	1.195(0.638–2.240)			
sex		0.789		
Female	1.0			
Male	0.919(0.496–1.704)			
Tumor location		0.177		
colon	1.0			
rectum	0.636(0.329–1.228)			
Differention		0.160		
well	1.0			
moderate	0.470(0.217–1.019)	0.056		
poor	0.643(0.081–5.080)	0.675		
Lymphnode Examined		**0.046**		**0.006**
>12	1.0		1.0	
<=12	1.874(1.012–3.471)		2.500(1.304–4.795)	
Tumor diameter		0.734		
<5 cm	1.0			
>=5 cm	1.115(0.595–2.090)			
TNM-stage		0.101		
I	1.0			
II	4.137(0.963–17.765)	0.056		
III	4.891(1.142–20.941)	0.032		
KRAS status		0.924		
wt	1.0			
mutant	1.030(0.558–1.904)			
NRAS status		**0.024**		**0.003**
wt	1.0		1.0	
mutant	3.280(1.167–9.219)		5.152(1.758–15.101)	
BRAF status		0.323		
wt	1.0			
mutant	2.048(0.494–8.499)			
PIK3CA status		**0.038**		**0.003**
wt	1.0		1.0	
mutant	2.131(1.044–4.352)		3.129(1.463–6.693)	

### Data availability statement

The datasets generated during and/or analyzed during the current study are available from the corresponding authors.

## Results

### Mutation frequencies and distributions

*KRAS* mutations were detected in 186 out of 353 (52.7%) tumor samples, of which 149 (42.2%) had mutations in exon 2, 8 (2.3%) in exon 3, and 29 (8.2%) in exon 4. Among mutations in *KRAS* exon 2, 109 (30.9%) had single mutations in codon 12 and 38 (10.8%) in codon 13. The main mutant type was 35G>A (G12D, 27.9%), followed by 38G>A (G13D, 18.8%). The most frequently mutation type in exon 3 was 183A>C (Q61H), but 176C>A (A59E), 181C>A (Q61K), 182A>T (Q61L) and 182A>G (Q61R) were also found in our study. In exon 4, the most common mutation was 436G>A (A146T), followed by 351A>T (K117N), 437C>T (A146V), 350A>G (K117R), 436G>C (A146P) and 441G>T (K147N). Moreover, one case harbored both G12V and G12D, while another had both G13D and D54N. *NRAS* mutations were identified in 12 out of 353 (3.4%) tumor samples, with 5 cases in exon 2 (1.4%) and 7 cases in exon 3 (2.0%). The main mutant types were 35G>A (G12D) in exon 2 and 181C>A (Q61K) in exon 3.

Sixteen (4.5%) patients harbored *BRAF* exon 15 mutations, with 14 mutations in codon 600 and 2 mutations in codon 601. The most common mutation was 1799T>A (V600E). *PIK3CA* mutations could not be detected in three samples, 46 out of 350 patients (12.3%) harbored *PIK3CA* mutations, with 26 mutations in exon 9 (7.4%) and 20 mutations in exon 20 (5.7%). The most frequent mutant types were 1633G>A (E545K) in exon 9 and 3140A>G (H1047R) in exon 20. Mutations are summarized in Table 3.

**Table 3:** Frequency and distribution of *KRAS*, *NRAS*, *BRAF* and *PIK3CA* mutations *DNA of three samples was not available for PIK3CA exon 20.

	Nucleotide	Amino acid	Case(%)		Nucleotide	Amino acid	Case(%)
KRAS exon 2			149(42.2%)	**NRAS exon 2**			5(1.4%)
	34G>A	G12S	5		34G>C	G12R	1
	34G>T	G12C	9		35G>C	G12A	1
	34G>C	G12R	1		35G>A	G12D	2
	35G>A	G12D	52		38G>A	G13D	1
	35G>C	G12A	9	**NRAS exon 3**			7(2%)
	35G>T	G12V	33		178G>A	G60R	1
	35G>T&35G>A	G12V&G12D	1		181C>A	Q61K	6
	37G>C	G13R	1	**BRAF exon 15**			16(4.5%)
	37G>T	G13C	2		1799T>A	V600E	14
	38G>A	G13D	35		1801A>G	K601E	1
	38G>A&160G>A	G13D&D54N	1		1803A>C	K601N	1
KRAS exon 3			8(2.3%)	**PIK3CA exon 9**			26(7.4%)
	176C>A	A59E	1		1624G>A	E542K	5
	181C>A	Q61K	1		1633G>A	E545K	11
	182A>T	Q61L	1		1634A>C	E545A	2
	182A>G	Q61R	1		1635G>C	E545D	1
	183A>C	Q61H	4		1636C>A	Q546K	5
KRAS exon 4			29(8.2%)		1637A>G	Q546R	1
	351A>T	K117N	3		1638G>T	Q546H	1
	350A>G	K117R	1	PIK3CA exon 20*			20(5.7%)
	436G>A	A146T	21		3062A>T	Y1021F	2
	436G>C	A146P	1		3073A>G	T1025A	1
	437C>T	A146V	2		3129G>A	M1043I	1
	441G>T	K147N	1		3139C>T	H1047Y	2
					3140A>G	H1047R	11
					3140A>T	H1047L	1

**Figure 2:**
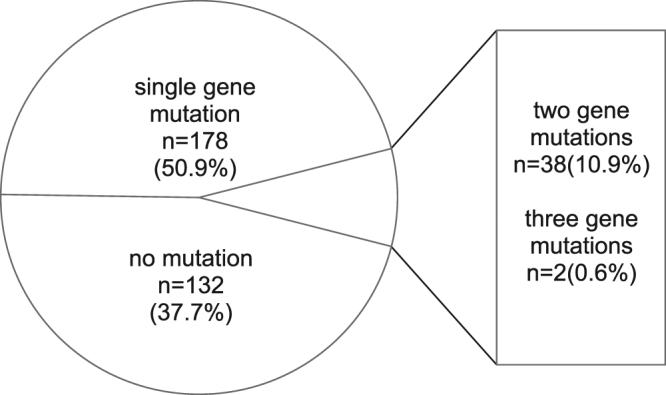
The distribution of mutations is illustrated in a pie chart of 350 colorectal cancer samples.

In total, 197 patients (55.8%) had *RAS* mutations. 49 patients (13.9%) had mutations outside *KRAS* exon 2. 218 patients (62.3%) carried one or more mutations, of which 178 (50.9%) harbored a single gene mutation, 38 patients (10.9%) two gene mutations, and 2 patients (0.6%) three gene mutations. In patients carrying two mutations, 34 patients had mutations in both *KRAS* and *PIK3CA* and 2 patients in both *BRAF* and *PIK3CA*. *BRAF* and *KRAS* exon 2 mutations were mutually exclusive, but we identified one patient who had concomitant *KRAS* exon 4 and *BRAF* mutations and one patient who had both *NRAS* and *BRAF* mutations. In addition, one patient suffered from *KRAS*, *BRAF* and *PIK3CA* mutations, while another patient harbored two *KRAS* and one *PIK3CA* mutation. The mutation distribution is shown in Fig. 2 and Supplementary Table S3.

### Clinicopathological characteristics of mutations

We did not find any significant correlation between *KRAS* (exon 2, 3 and 4) mutations and patients’ clinicopathological characteristics (Table 1). Female patients harbored more *NRAS* (exon 2 and 3) mutations than male patients (75.0% vs 25.0%; P = 0.019). Compared to *BRAF* wt patients, patients with *BRAF* mutations were more likely to exhibit poor differentiation (P = 0.030), advanced TNM stage (P = 0.001), larger/more invasive tumor (P = 0.019), higher lymph node metastasis rate (P = 0.004), and higher synchronous (P = 0.002) and metachronous metastasis rate (P = 0.016). *PIK3CA* mutations occurred more frequently in colon than in rectal cancers (P = 0.014). Those who had at least one mutation, occurred more frequently among older patients (average age: 67.2 vs 64.4 years old, P = 0.04). There were no significant associations in clinicopathological characteristics between double gene mutant and wt patients (see Supplementary Table S4).

We then investigated the associations between different subtypes of *KRAS* mutations and patients’ clinicopathological characteristics. *KRAS* exon 2 mutation appeared more frequent in older patients (average age: 67.7 years old vs 64.9 years old; P = 0.036) and was associated with higher lymph node metastasis rate (52.3% vs 41.6%; P = 0.046). *KRAS* exon 3 mutation was more likely to appear in lower TNM stage (P = 0.011) and smaller/less invasive tumor (P = 0.001) patients. Data are shown in Supplementary Table S5.

### Survival analysis

**Figure 3:**
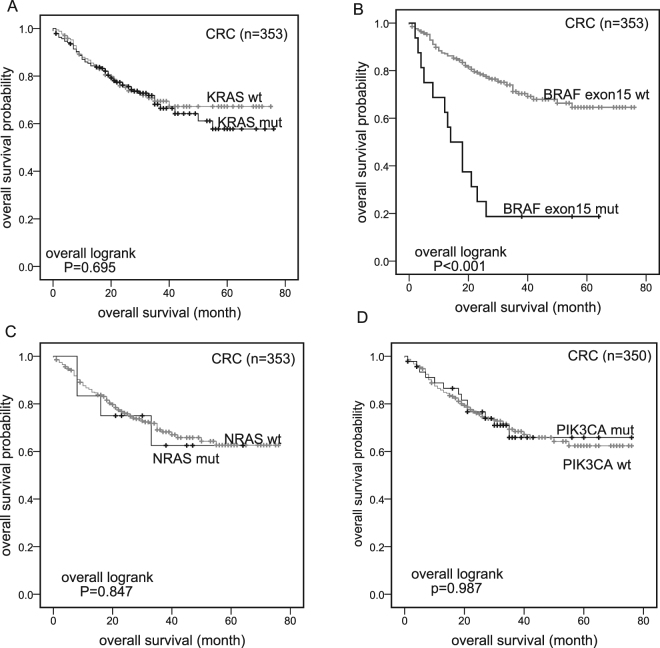
Kaplan-Meier curves. OS since surgery for patients with (black) and without (gray) mutations in 353 CRC patients. Panel D: DNA of three samples was not available for PIK3CA mutation analysis. wt: Wild-type; mut: Mutant.

**Figure 4:**
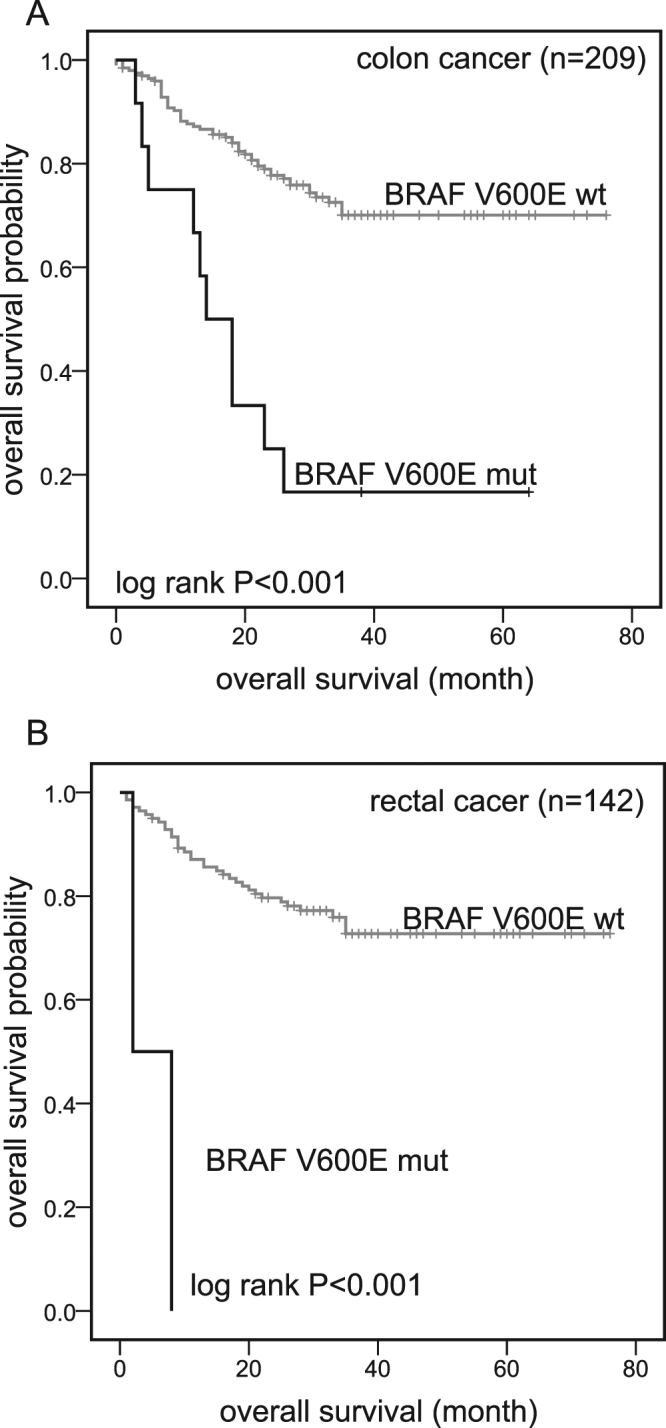
Kaplan-Meier curves. OS since surgery for patients with (black) and without (gray) *BRAF* V600E mutations in colon or rectal cancer. (**A**) 3-year OS in *BRAF* V600E mut versus *BRAF* wt colon cancer patients: 16.7% versus 74.1%; log-rank P < 0.001. One colon cancer patient harboring a *BRAF* K601E mutation was excluded in this analysis. (**B**) 3-year OS in *BRAF* V600E mut versus *BRAF* wt rectal cancer patients: 0% versus 75.7%; log-rank P < 0.001. One rectal cancer patient harboring a *BRAF* K601N mutation was excluded in this analysis. wt: Wild-type; mut: Mutant.

KM analysis showed no difference of OS between *KRAS-*, *NRAS*- or *PIK3CA*-mutant patients and wt patients (P = 0.695; P = 0.847; P = 0.987; Fig. 3A,C,D). Only *BRAF* mutations had a strong association with poorer OS (3-year OS) in *BRAF-*mutant vs *BRAF-*wt patients (14.3% vs 74.6%; P < 0.001; Fig. 3B). In our previous study, *BRAF* V600E mutations were found only in colon cancers and strongly revealed poorer OS in colon patients. In this extended study, we found *BRAF* V600E mutations had poorer prognosis both in colon and rectal cancer patients (Fig. 4A,B). Because only two *BRAF* V600E-mutant patients with rectal cancer were included, the result still needs to be confirmed in the future.

Because *BRAF* mutations confer a poorer prognosis, several recent studies have suggested to exclude *BRAF*-mutant patients from *KRAS*-wt patients when assessing the prognostic value of *KRAS*^17,21,22^. After we excluded *BRAF-*mutant patients, a total of 337 patients (353 patients - 16 *BRAF-*mutant patients) remained in the analysis (see Supplementary Fig. S1A). OS between *KRAS-*mutated and wt patients was still not different (P = 0.142). Likewise, there were also no differences in OS between *NRAS-* (P = 0.524) or *PIK3CA-* (P = 0.658) mutated and wt patients (data not shown). Finally, we observed no significant correlations between OS and the three subtypes of *KRAS* mutations.

**Figure 5:**
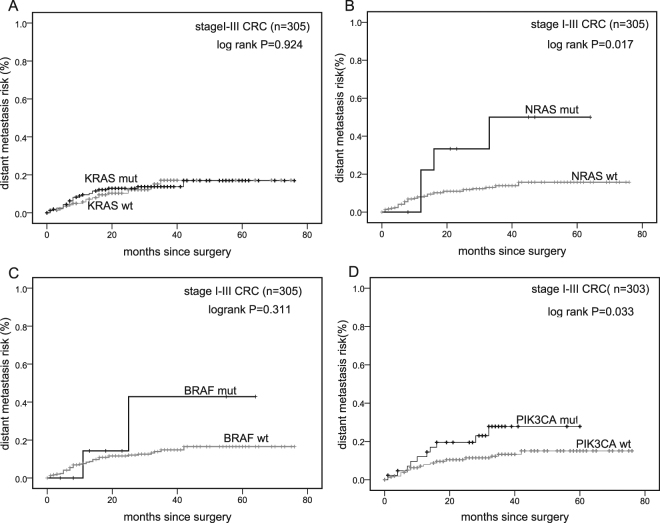
Kaplan-Meier curves. Distant metastasis rates since surgery for 305 stage I to III patients with (black) and without (gray) mutations. Panel (**D**) DNA of two samples were not available for PIK3CA mutation analysis. wt: Wild-type; mut: Mutant.

Previous studies showed that *PIK3CA* mutations were strongly correlated with a higher local recurrence rate in stage I to III rectal cancer patients, but did not find the correlation with distant metastases^15^. In our present study, local recurrence analysis was not feasible because of a lack of relevant data. Instead, we analyzed the relationship between different mutations and the occurrence of postoperative distant metastases. In total, 305 patients (353 patients −45 patients with stage IV disease and 3 patients without proper staging) were included in the analysis (see Supplementary Fig. S1B) In this group, 41 patients developed postoperative distant metastases. Patients with *PIK3CA* mutations had a 2-fold increased distant metastasis rate compared with *PIK3CA* wt patients (3-year risks, 23.3% vs 11.5%, P = 0.03) and a shorter interval between surgery and the diagnosis of a metastasis (mean metastasis-free intervals: 67.1 months vs 47.6 months; P = 0.033). More intriguingly, patients with *NRAS* mutations showed a > 3-fold increase in distant metastases and a shorter metastasis-free interval (3-year risks, 40.0% vs 12.2%, P = 0.012; mean metastasis-free intervals: 66.6 months vs 41.9 months, P = 0.017). No correlations were found between other mutations and distant metastases (Fig. 5).

In univariate Cox regression analysis for distant metastases, the variables including age, sex, tumor location, differentiation grade, number of lymph nodes examined, tumor diameter, TNM stage and *KRAS*/*NRAS*/*BRAF*/*PIK3CA* mutations that are listed in Table 2 were examined. Besides numbers of lymph nodes examined (P = 0.046), *PIK3CA* (hazard ratio (HR), 2.131; 95% confidence interval (CI), 1.044–4.352; P = 0.038) and *NRAS* mutations (HR, 3.280; 95%CI, 1.167–9.219; P = 0.024) revealed a higher risk of distant metastases. The three variables were entered into a multivariate analysis with stepwise backward elimination. *PIK3CA* (HR, 3.129; 95% CI, 1.463–6.693; P = 0.003) and *NRAS* (HR, 5.152; 95% CI, 1.758–15.101; P = 0.003) mutations both persisted as prognostic markers for distant metastases in stage I to III patients. No significant interactions were observed between the variables.

## Discussion

In this study, we identified that 13.9% (49 out of 353) CRC patients carried mutations at *RAS* exons outside the *KRAS* exon 2. The mutations were mainly located in exons 3 and 4 of *KRAS*, and in exons 2 and 3 of *NRAS* genes. More importantly, we found that stage I to III patients who carried *PIK3CA-* or *NRAS-*mutated genes had a higher risk to develop distant metastases after surgery and had shorter metastasis-free intervals.

Mutations of *KRAS* exon 2 were most common in 353 Chinese CRC patients and were typically located in codons 12 and 13. *BRAF* mutation frequency was 4.5% in CRCs and substantially lower than in Western countries (10–15%)^23^, but was consistent with other Asian regions, such as Japan^21^ and Taiwan^24^. The *PIK3CA* mutation occurred in 13.1% of CRCs in our study. All mutation frequencies found were consistent with our previous study^20^.

Mutations of *RAS* exons outside *KRAS* exon 2 occurred in 13.9% of CRC patients in our study, of which 8 (2.5%) had single mutations in *KRAS* exon 3, 29 (8.2%) in *KRAS* exon 4, and 12 (3.4%) in *NRAS* exon 2 or 3. Among the 8 mutations in *KRAS* exon 3, 7 mutations occurred in codon 61, which is similar to another Chinese study^11^. Mutations in exon 4 were mainly located in codons 117 and 146. The mutation frequency in codon 146 (6.8%) was higher than the observed rate in Western countries, but similar to the reported frequencies in Hong Kong (5.6%, 7/126)^3,25,26^. *NRAS* mutations were identified in 12 (3.4%) out of 353 tumor samples. The mutation frequency of *NRAS* exon 3 (7, 2.0%) was slightly higher than in *NRAS* exon 2 (5, 1.4%) and similar to Shen’s study of the Chinese population^11^.

Among recent Western studies, one showed that 12.1% of 513 *KRAS* exon 2 wt American CRCs had mutations in either *KRAS* exon 3 or 4, or *NRAS* exon 2 or 3^27^. Another study^9^ in 639 *KRAS* exon 2 wt European CRCs found that these *RAS* mutations reached a prevalence of 17%. In our study, mutation frequencies of *RAS* exons outside *KRAS* exon 2 reached approximately 14% as well. These frequencies are also close to other Chinese studies published in recent years with the exception of the mutation frequency of *KRAS* exon 4^11,28,29^. The mutation frequency in *KRAS* exon 4 (8.2%) in our study was higher than the frequencies in two Western studies mentioned (1.9% and 3.3%), but close to two Chinese reports (one study: 5.6% of 126 Hong Kong CRCs; the other study: 4.1% of 1110 Chinese CRCs)^3,28^. However, another study reported that only 2.7% of 1506 Hong Kong CRCs had mutation in *KRAS* exon 4. At present, we have no explanation for this discrepancy. We used tumor samples from two hospitals in different regions and studied only patients without preoperative radiotherapy or chemotherapy. Furthermore, our samples were tested for all *RAS* mutations regardless of whether they harbored *KRAS* exon 2 mutations. These boundary conditions enabled us to make a better estimate of mutation frequencies in *RAS* exons outside *KRAS* exon 2 in Chinese CRCs.

In western countries a mutation test for *KRAS* exon 2 is routine clinical practice to qualify for anti-EGFR treatment, but more than half of the *KRAS* exon 2 wt patients are, nonetheless, resistant to this treatment^6^. Therefore, it is necessary to further extend the genetic status in *KRAS* exon 2 wt patients. In agreement, a large proportion of clinical trials has demonstrated mutations at *RAS* sequences outside *KRAS* exon 2. These mutations, which are called “rare mutations” in literature, confer a detrimental effect on the response to anti-EGFR MoAbs^7,8,23,30^. Because most of the data came from Western countries, it was important to establish the mutation distribution and frequencies of these rare mutations in the Chinese population. In our study, we found that up to 14% CRC patients carried these rare mutations, and that these patients represent almost a quarter (24.0%, 48 out of 204 (353 CRCs-149 *KRAS* exon 2 mutants)) among the *KRAS* exon 2 wt patients.

More than 60% of cases carried either one or more activating mutations in the RAS-RAF or the PI3K-PTEN-AKT pathways in our study-cohort, so that they may well be resistant to anti-EGFR MoAbs. Irrespective of *KRAS* exon 2 mutations, it remains controversial whether it is important to investigate these rare mutations in clinical practice. Considering there are a series of rare mutation subtypes in the two pathways, it is hard to test every mutation site. Although the total mutation frequency of these rare mutations in our study was similar to Western countries, there was a difference in the frequency of each mutation subtype, as best shown for *KRAS* exon 4. Therefore, our findings can contribute to determine frequent mutation sites for priority detection in the Chinese population.

At first, we found no associations between *KRAS* mutations (including exons 2, 3 and 4) and patients’ clinicopathological features. However, each mutation subtype confers unique conformational or structural alterations, which may confer variable effects on tumor progression^31,32^. To verify this view, we further analyzed *KRAS* mutations at different exons. And found *KRAS* exon 2 mutations occurred more frequently in older patients and patients with lymph-node metastases.

*KRAS* exon 3 mutations seemed to confer a less aggressive biological behavior, because they were associated with lower TNM stage and smaller/less invasive tumor. Considering there were only 9 patients harboring *KRAS* exon 3 mutations and those 9 patients contained 5 subtypes, this finding does not yet allow us to draw a firm conclusion.

We found that *NRAS* mutations occurred more frequently in female patients (75.0% vs 41.1%). Other studies in Chinese populations did not find any correlations between NRAS mutations and clinicopathological characteristics^28^. Because we observed only 5 cases with mutations in *NRAS* exon 2 and only 7 cases in exon 3, we could not determine their clinical impact.

Mutations in *PIK3CA* may be significantly related to relapse-free survival in stage II and III patients, but the sample size was small (n = 96)^33^. In 450 stage I to III colon cancers, *PIK3CA* mutation was associated with a significant increase in colon cancer–specific mortality in the *KRAS* wt patients^14^. A recent study on Chinese patients showed that among *KRAS*, *BRAF*, *PIK3CA* and *NRAS* mutations in stage II to III colon cancers (n = 228), only *PIK3CA* mutations were an independent prognostic biomarker for poor OS among stage III patients^34^. In agreement, we found that patients with *PIK3CA* mutations had a 2-fold increased distant metastasis rate and shorter metastasis-free intervals, compared with the *PIK3CA* wt patients in stages I-III. In the following univariate and multivariate Cox regression analysis, *PIK3CA* mutations persisted as an independent prognostic marker for distant metastasis among stage I to III patients when mixed with other confounding factors. This observation was consistent with the above mentioned studies, but we had a relative large sample size.

None of the previous studies investigated the prognostic value of the rare mutations among stage I to III patients. In our study, a *NRAS* mutation acted as an independent prognostic marker for distant metastasis (3-fold increased rate) in stage I to III patients, with shorter metastasis-free intervals than *NRAS* wt patients. In fact, a large study of patients with in metastatic CRCs (n = 786) showed recently that *NRAS* mutations were associated with shorter OS^19^, but the effect of such mutations in stage I to III patients was not discussed. To the best of our knowledge, we are, therefore, the first to illustrate the prognostic role of *NRAS* mutations on distance metastases in early, mid-stage patients. Nevertheless, this observation requires further confirmation in a larger population of Chinese patients given the relatively low frequencies of *NRAS* mutations (n = 12) in our data.

*KRAS* and *NRAS* proteins are homologous enzymes of the *RAS* protein family^35^ and genetic studies have clarified apparent differences between *RAS* isoforms. Furthermore, *KRAS* and *NRAS* mutations have a mutually exclusive mechanism to induce carcinogenesis^36^. Mutant *KRAS* induces excessive proliferation and differentiation of human colonic epithelial cell lines, whereas mutant *NRAS* suppresses apoptosis^37^. Accordingly, mutant *NRAS* signals *in vivo* through the non-canonical RAS-RAF-MEK-STAT3 (Signal transducer and activator of transcription 3) MAPK pathway to regulate cell apoptosis. We speculate that the ability to predict distance metastasis based on *NRAS* mutations may be responsible for their distinct tumorigenic mechanism.

The limitations of our study are its relatively small sample size, retrospective nature, short follow-up time, and lack of epigenetic or MSI status, which are important for risk assessment of CRC. *NRAS* mutation frequency was too low to analyze its mutation subgroups. We will, therefore, enlarge our sample size by recruiting more CRC patients from other clinical centers. On the other hand, we collected tumor samples from two hospitals in different regions and included patients who had not received preoperative radiotherapy or chemotherapy. These aspects make our data more representative and prognostic for new patients.

## Conclusion

In conclusion, we studied mutations in the RAS-RAF and PI3K-PTEN-AKT pathways, especially the frequencies and distributions of so-called rare mutations, including *KRAS* exons 3 and 4, and *NRAS* exons 2 and 3 in 353 Chinese CRC patients. Among the mutations, *PIK3CA* and *NRAS* mutations had prognostic value and predicted distant metastasis in stage I to III CRC patients. Our findings show that these new molecular features may be important for better decision making in clinical practice for postoperative chemotherapy, such as the use of anti-EGFR MoAbs and/or radiotherapy as postoperative adjuvant therapy. The *NRAS* mutation is promising for future studies because of its unique carcinogenic mechanism and biological characteristics.

## Electronic supplementary material

Supplementary Information
