# Supplementary material for: Mutation status and prognostic values of KRAS, NRAS, BRAF and PIK3CA in 353 Chinese colorectal cancer patients
Source: Sci Rep. 2018 Apr 17;8:6076. doi: 10.1038/s41598-018-24306-1 (PMC5904111; doi:10.1038/s41598-018-24306-1)
Supplement: Supplementary file 1 — Supplementary Information [file 41598_2018_24306_MOESM1_ESM.doc]

# Mutation status and prognostic values of *KRAS*, *NRAS*, *BRAF* and *PIK3CA* in 353 Chinese colorectal cancer patients

Fang Guo^1^, Hai Gong^2^, Huanhuan Zhao^3^, Jing Chen^1^, Yiming Zhang^1^, Lihua Zhang^4^, Xin Shi^5^, Aifeng Zhang^4^, Hui Jin^6^, Jianqiong Zhang^7*^, Youji He^1*^

|  |  | **No. patients** | | |
| --- | --- | --- | --- | --- |
|  |  | N=353 | N=286 | p value |
| **sex** | **male** | 204 | 172 | 0.548^a^ |
|  | **female** | 149 | 114 |  |
| **Age** | **age** | 66.08 | 67.21 | 0.267^c^ |
| **Location** | **colon** | 210 | 156 | 0.209^a^ |
|  | **rectum** | 143 | 130 |  |
| **Differentiation** | **well** | 43 | 34 | 0.717^b^ |
|  | **moderate** | 271 | 177 |  |
|  | **poor** | 13 | 11 |  |
|  | **missing** | 26 | 64 |  |
| **Tumor diameter** | **<5cm** | 171 | 116 | 0.583^a^ |
|  | **>=5cm** | 179 | 133 |  |
|  | **missing** | 3 | 37 |  |
| **TNM-stage** | **0** | 0 | 4 | 0.570^b^ |
|  | **I** | 53 | 38 |  |
|  | **II** | 125 | 84 |  |
|  | **III** | 131 | 105 |  |
|  | **IV** | 36 | 16 |  |
|  | **missing** | 8 | 39 |  |
| **T** | **T1** | 5 | 14 | 0.939^b^ |
|  | **T2** | 69 | 33 |  |
|  | **T3** | 256 | 187 |  |
|  | **T4** | 18 | 9 |  |
|  | **missing** | 5 | 43 |  |
| **N** | **N(-)** | 188 | 133 | 0.853^a^ |
|  | **N(+)** | 159 | 116 |  |
|  | **missing** | 6 | 37 |  |
| a:chi-square test; b:Mann-Whitney test; c:t test | | | | |

**Supplementary Table S1** A summary of the clinicopathological characteristics of the patients included and excluded in this study.

| **Genes** | **Primers (sequence 5’– 3’)** | **Genes** | **Primers (sequence 5’– 3’)** |
| --- | --- | --- | --- |
| ***KRAS*-Exon 2** | **F:**GGTACTGGTGGAGTATTTGATAG | ***NRAS*-Exon 3** | **F:**CCCCTTACCCTCCACACC |
|  | **R:**ATTAGCTGTATCGTCAAGGCACT |  | **R:**TCGCCTGTCCTCATGTATTG |
| ***KRAS*-Exon 3** | **F:**TGCACTGTAATAATCCAGACTGTG | ***BRAF*-Exon15** | **F:**TGAAGACCTCACAGTAAAAATAGGTG |
|  | **R:**CATGTACTGGTCCCTCATTGC |  | **R:**GGCCAAAAATTTAATCAGTGGA |
| ***KRAS*-Exon 4** | **F:**CTGAAGATGTACCTATGGTCCTAGT | ***PIK3CA*-Exon 9** | **F:**AGTAACAGACTAGCTAGAGACAAT |
|  | **R:**TTATTTCAGTGTTACTTACCTGTCTTG |  | **R:**CCATTTTAGCACTTACCTGTGAC |
| ***NRAS*-Exon 2** | **F:**GATGTGGCTCGCCAATTAAC | ***PIK3CA*-Exon20** | **F:**TCTTTTGATGACATTGCATACAT |
|  | **R:**AAGTGGTTCTGGATTAGCTGGA |  | **R:**TCCAGAGTGAGCTTTCATTTTC |

**Supplementary Table S2** The primers used in PCR amplification and sequencing

| **Mutation type** | **Case (40)** |
| --- | --- |
| KRAS exon 2&PIK3CA | 24 |
| KRAS exon 3&PIK3CA | 1 |
| KRAS exon 4&PIK3CA | 9 |
| NRAS exon 2&BRAF | 1 |
| KRAS exon 4&BRAF | 1 |
| BRAF & PIK3CA | 2 |
| KRAS exon 2&exon 3&PIK3CA | 1 |
| KRAS exon 4&BRAF&PIK3CA | 1 |

**Supplementary Table S3** Distribution of two or three concomitant gene mutations

|  |  | **case (170)** | **PI3K pathway wild type,**  **n(%)** | **2 mut in PI3K pathway n (%)** | **p value** |
| --- | --- | --- | --- | --- | --- |
| **sex** | **male** | **69** | 53 (40.2) | 16 (42.1) | 0.829^a^ |
|  | **female** | **101** | 79 (59.8) | 22 (57.9) |  |
| **Age** |  |  | 64.44 | 64.16 | 0.910^d^ |
| **Location** | **colon** | **108** | 79 (59.8) | 29 (76.3) | 0.063^a^ |
|  | **rectum** | **62** | 53 (40.2) | 9 (23.7) |  |
| **Differentiation** | **well** | **23** | 18 (13.6) | 5 (13.2) | 0.914^c^ |
|  | **moderate** | **130** | 102 (77.3) | 28 (73.7) |  |
|  | **poor** | **8** | 6 (4.5) | 2(5.3) |  |
|  | **missing** | **9** | 6 (4.5) | 3 (7.9) |  |
| **Tumor diameter** | **<5cm** | **77** | 62 (47.0) | 15 (39.5) | 0.488^a^ |
|  | **>=5cm** | **92** | 70 (53.0) | 22 (57.9) |  |
|  | **missing** | **1** | 0 (0) | 1 (2.6) |  |
| **TNM-stage** | **I** | **23** | 17(12.9) | 6(15.8) | 0.880^c^ |
|  | **II** | **69** | 56(42.4) | 13(34.2) |  |
|  | **III** | **58** | 42(31.8) | 16(42.1) |  |
|  | **IV** | **18** | 15(11.4) | 3(7.9) |  |
|  | **missing** | **2** | 2(1.5) | 0(0) |  |
| **T** | **T1** | **2** | 2 (1.5) | 0 (0) | 0.930^c^ |
|  | **T2** | **32** | 24 (18.2) | 8 (21.1) |  |
|  | **T3** | **123** | 96 (72.7) | 27 (71.1) |  |
|  | **T4** | **11** | 8 (6.1) | 3 (7.9) |  |
|  | **missing** | **2** | 2 (1.5) | 0 |  |
| **N** | **N(-)** | **98** | 78 (59.1) | 20 (52.6) | 0.447^a^ |
|  | **N(+)** | **71** | 53 (40.2) | 18 (47.4) |  |
|  | **missing** | **1** | 1 (0.8) | 0 |  |
| **M-synchronous** | **M(-)** | **151** | 116(87.9) | 35(92.1) | 0.388^b^ |
|  | **M(+)** | **18** | 15(11.4) | 3(7.9) |  |
|  | **missing** | **1** | 1(0.8) | 0(0) |  |
| **M-metachronous** | **M(-)** | **147** | 117(88.6) | 30(78.9) | 0.124^a^ |
|  | **M(+)** | **23** | 15(11.4) | 8(21.1) |  |

**Supplementary Table S4** Clinicopathological characteristics between patients carrying two gene mutations and a wild-type PI3K pathway. a: chi-square test; b: Fisher exact test; c: Mann-Whitney test; d: t test.

|  | | **case** | **KRAS exon 2** | | | **KRAS exon 3** | | | **KRAS exon 4** | | |
| --- | --- | --- | --- | --- | --- | --- | --- | --- | --- | --- | --- |
|  |  | 353 | No, n (%) | Yes, N (%) | p value | No, n (%) | Yes, n (%) | p value | No, n (%) | Yes, n (%) | p value |
| **sex** | **Male** | 204 | 117 (57.4) | 87 (58.4) | 0.846^a^ | 144 (41.9) | 5(55.6) | 0.502^a^ | 136(42.0) | 13(44.8) | 0.766^a^ |
|  | **Female** | 149 | 87 (42.6) | 62 (41.6) |  | 200(58.1) | 4(44.4) |  | 188 (58.0) | 16(55.2) |  |
| **Age** |  |  | 64.89 | 67.71 | **0.036^d^** | 66.13 | 64.33 | 0.671^d^ | 66.21 | 64.66 | 0.522^d^ |
| **Location** | **Colon** | 210 | 126 (61.8) | 84 (56.4) | 0.308^a^ | 205 (59.6) | 5 (55.6) | 1.000^b^ | 194 (59.9) | 16(55.2) | 0.621^a^ |
|  | **rectum** | 143 | 78 (38.2) | 65 (43.6) |  | 139(40.4) | 4 (44.4) |  | 130(40.1) | 13 (44.8) |  |
| **Differentiation** | **Well** | 44 | 29 (14.2) | 15 (10.1) | 0.818^c^ | 42(12.2) | 2 (22.2) | 0.207^c^ | 39 (12.0) | 5 (17.2) | 0.058^c^ |
|  | **Moderate** | 274 | 148 (72.5) | 126 (84.6) |  | 267 (77.6) | 7 (77.8) |  | 259 (79.9) | 15 (51.7) |  |
|  | **Poor** | 12 | 12 (5.9) | 0 |  | 12 (3.5) | 0 (0) |  | 9(2.8) | 3(10.3) |  |
|  | **Missing** | 23 | 15 (7.4) | 8 (5.4) |  | 23 (6.7) | 0 (0) |  | 17 (5.2) | 6(20.7) |  |
| **Tumor diameter** | **<5cm** | 171 | 92 (45.1) | 79 (53.0) | 0.120^a^ | 169(49.1) | 2(22.2) | 0.175^b^ | 160 (49.4) | 11(37.9) | 0.291^a^ |
|  | **>=5cm** | 179 | 111 (54.4) | 68 (45.6) |  | 172 (50.0) | 7(77.8) |  | 162 (50.0) | 17(58.6) |  |
|  | **Missing** | 3 | 1 (0.5) | 2 (1.3) |  | 3(0.9) | 0 |  | 2(0.6) | 1 (3.4) |  |
| **TNM stage** | **I** | 53 | 29(14.2) | 24(16.1) | 0.552^c^ | 48(14.0) | 5(55.6) | **0.011^c^** | 49(15.1) | 4(13.8) | 0.500^c^ |
|  | **II** | 126 | 81(39.7) | 45(30.2) |  | 124(36.0) | 2(22.2) |  | 116(35.8) | 10(34.5) |  |
|  | **III** | 126 | 64(31.4) | 62(41.6) |  | 124(36.0) | 2(22.2) |  | 117(36.1) | 9(31.0) |  |
|  | **IV** | 45 | 28(13.7) | 17(11.4) |  | 45(13.1) | 0(0) |  | 39(12.0) | 6(20.7) |  |
|  | **Missing** | 3 | 2(1.0) | 1(0.7) |  | 3(0.9) | 0(0) |  | 3(0.9) | 0(0) |  |
| **T** | **T1** | 5 | 4 (2.0) | 1 (0.7) | 0.614^c^ | 4 (1.2) | 1(11.1) | **0.001^c^** | 4 (1.2) | 1 (3.4) | 0.889^c^ |
|  | **T2** | 69 | 37 (18.1) | 32 (21.5) |  | 64 (18.6) | 5 (55.6) |  | 64 (19.8) | 5 (17.2) |  |
|  | **T3** | 258 | 149 (73.0) | 109 (73.2) |  | 255 (74.1) | 3 (33.3) |  | 237 (73.1) | 21 (72.4) |  |
|  | **T4** | 19 | 12 (5.9) | 7 (4.7) |  | 19 (5.5) | 0 (0) |  | 17 (5.2) | 2 (6.9) |  |
|  | Missing | 2 | 2 (1.0) | 0 |  | 2 (0.6) | 0 (0) |  | 2 (0.6) | 0 (0) |  |
| **N** | **N(-)** | 189 | 118 (58.4) | 71 (47.7) | **0.046^a^** | 182(52.9) | 7 (77.8) | 0.186^b^ | 174(53.7) | 15 (51.7) | 0.976^a^ |
|  | **N(+)** | 162 | 84 (41.6) | 78 (52.3) |  | 160(46.5) | 2 (22.2) |  | 149(46.0) | 13 (44.8) |  |
|  | **Missing** | 2 | 2 (1.0) | 0 |  | 12(0.6) | 0(0) |  | 1 (0.3) | 1 (3.4) |  |
| **M-synchronous** | **(-)** | 306 | 175(85.8) | 131(87.9) | 0.523^a^ | 297(86.3) | 9(100.0) | 0.611^b^ | 283(87.3) | 23(79.3) | 0.239^b^ |
|  | **(+)** | 45 | 28(13.7) | 17(11.4) |  | 45(13.1) | 0(0) |  | 39(12.0) | 6(20.7) |  |
|  | **missing** | 2 | 1(0.5) | 1(0.7) |  | 2(0.6) | 0(0) |  | 2(0.6) | 0(0) |  |
| **M-metachronous** | **(-)** | 300 | 173(84.8) | 127(85.2) | 0.911^a^ | 291(84.6) | 9(100.0) | 0.366^b^ | 276(85.2) | 24(82.8) | 0.785^b^ |
|  | **(+)** | 53 | 31(15.2) | 22(14.8) |  | 53(15.4) | 0(0) |  | 48(14.8) | 5(17.2) |  |

**Supplementary Table S5** Clinicopathological characteristics of KRAS mutations in different exons in 353 colorectal cancer patients. a: chi-square test; b: Fisher exact test; c: Mann-Whitney test; d: t test


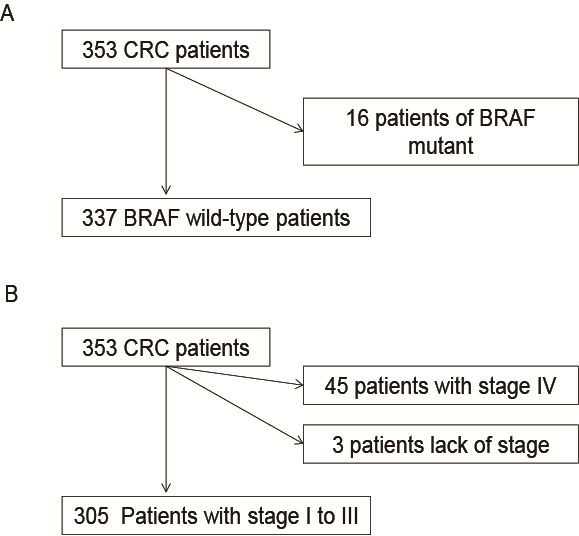


**Supplementary Fig.S1** Patient selection
